# Cycling-Induced Capacity Increase of Graphene Aerogel/ZnO Nanomembrane Composite Anode Fabricated by Atomic Layer Deposition

**DOI:** 10.1186/s11671-019-2900-7

**Published:** 2019-02-28

**Authors:** Dingrun Wang, Yalan Li, Yuting Zhao, Qinglei Guo, Siwei Yang, Guqiao Ding, YongFeng Mei, Gaoshan Huang

**Affiliations:** 1 Department of Materials Science, Fudan University, Shanghai, 200433 People’s Republic of China; 2 Center for Excellence in Superconducting Electronics (CENSE), State Key Laboratory of Functional Materials for Informatics, Shanghai Institute of Microsystem and information Technology, Chinese Academy of Science, Shanghai, 20050 People’s Republic of China

**Keywords:** Zinc oxide, Graphene aerogel, Atomic layer deposition, Capacity increase, Lithium-ion batteries

## Abstract

Zinc oxide (ZnO) nanomembranes/graphene aerogel (GAZ) composites were successfully fabricated via atomic layer deposition (ALD). The composition of GAZ composites can be controlled by changing the number of ALD cycles. Experimental results demonstrated that the anode made from GAZ composite with ZnO nanomembrane of 100 ALD cycles exhibited highest specific capacity and best rate performance. A capacity increase of more than 2 times during the first 500 cycles was observed, and a highest capacity of 1200 mAh g^−1^ at current density of 1000 mA g^−1^ was observed after 500 cycles. On the basis of detailed electrochemical investigations, we ascribe the remarkable cycling-induced capacity increase to the alloying process accompanied by the formation of a polymer layer resulting from kinetically activated electrolyte degradation at low voltage regions.

**Electronic supplementary material:**

The online version of this article (10.1186/s11671-019-2900-7) contains supplementary material, which is available to authorized users.

## Background

Lithium-ion batteries (LIBs) have been the dominant power source for consumer electronics due to their safety, high energy density, and low self-discharge [1–4]. However, graphite carbon as the traditional anode material delivers a charge-discharge capacity of 372 mAh g^−1^, which is not the promising anode material for the upcoming electric vehicles. It is urgent to develop new anode materials with high specific capacity to satisfy the ever-increasing demand in electric vehicles. Non-graphitic carbons such as graphene [5, 6], transition metal oxides (ZnO [7, 8], Fe_2_O_3_ [9, 10], Co_3_O_4_ [11, 12], MnO_2_ [13]), and their composites [14–16] have been the promising substitutes for graphite as anode materials.

ZnO has attracted much attention which is attributed to its high theoretic capacity (978 mAh/g, nearly two times higher than that of graphite), high lithium-ion diffusion efficiency, low cost, and environmental friendliness [17, 18]. However, ZnO suffers from large volume expansion/contraction (~ 163%) and poor conductivity, thus leading to fast capacity fading and poor cycling performance [8, 19]. Various strategies have been promoted to solve these problems, including the use of ZnO nanostructures (nanorod arrays [20] and nanosheets [7]) and carbon-based composites [21, 22]. Zhao et al. [21] fabricated three-dimensional carbon/ZnO nanomembrane composite foam through an immersing process. The composites could maintain more than 92% of the initial capacity after 700 cycles at 2 A g^−1^ because of the flexibility of ZnO nanomembranes and the effective electron/ion transport through carbon foam. In our previous work, we also successfully synthesized ZnO/expanded graphite composite and it could deliver a capacity of 438 mAh g^−1^ at 200 mA g^−1^ after 500 cycles [23]. In addition, graphene is considered as an excellent anode material with outstanding chemical stability, flexibility, and conductivity [24]. Graphene aerogel (GA), the 3D architectures of assembled 2D graphene sheets, not only keeps the advantage of the unique structure of graphene sheets, but also possesses ultralow density, high and tunable porosity, excellent mechanical strength, and extraordinary adsorption properties [25, 26]. We consider that the unique 3D structure of GA combined with ZnO nanomembranes may have advantageous applications in anodes for LIBs.

Herein, we designed an electrode structure with 3D GA coated with ZnO nanomembranes (GAZ). GA was firstly fabricated via a template-free, freeze-drying strategy and then coated with ZnO nanomembranes via atomic layer deposition (ALD) [25]. The components of GAZ composites can be easily tuned by changing the number of ALD cycles, which has been demonstrated in our previous researches [27–30]. In the composite, GA works as conductive skeletons and supports for ZnO nanomembranes. Its flexible nature helps to accommodate the volume change of ZnO during discharge/charge process, and the porous structure facilitates effective Li^+^ transport. Thus, when applied for lithium storage, the GAZ composites demonstrate high specific capacity and excellent rate performance; the composites deliver a reversible capacity of 1200 mAh g^−1^ at 1000 mA g^−1^ after 500 cycles. A notable capacity increase phenomenon was also observed in the charge-discharge process of the composites. Testing results confirm that the cycling-induced capacity increase can be attributed to the formation of a polymer layer in low voltage regions. We believe that the mechanism can be utilized to explain the similar phenomenon in other metal oxides.

## Methods

### Synthesis of GA

Graphene oxide (GO) used in this work was prepared from natural graphite using a modified Hummers’ method [25]. All chemicals were obtained from Sinopharm Chemical Reagent Co. Ltd., China. In a typical procedure for preparing the graphene hydrogel, 5.0 mg dopamine was added into the GO water dispersion followed by vigorous stirring for 10 min to obtain uniform solution. Fifteen milligrams l-ascorbic acid was added into the mixture with vigorous magnetic stirring until it was completely dissolved. Thirdly, the mixture was sealed in a glass vessel and heated at 95 °C for 10 h to transform the brown aqueous solution into a black graphene hydrogel. Next, the hydrogel was placed on a metal plate, which in turn rested in a pool of liquid nitrogen after dialysis in water to remove soluble species. The hydrogel was totally frozen by directional freezing from the metal-hydrogel interface to the top surface. And then, the aerogel was obtained from the frozen hydrogel by freeze-drying. The dry aerogel was placed in a glass vessel filled with perfluorooctyltriethoxysilane (PFOES)/ethanol (2 wt.%) with no direct contact between the liquid and the aerogel. Finally, the sealed glass vessel was heated at 70 °C for 8 h. GA could be obtained after thorough drying in air.

### Preparation of GAZ Composite

The obtained GA was coated with ZnO nanomembranes in the ALD chamber with dimethylzinc and deionized water as zinc and oxidant sources, respectively. The chamber temperature during deposition period was 150 °C. A typical ALD cycle includes diethylzinc pulse (30 ms), waiting time (3 s), and nitrogen (N_2_) purge (15 s) and water pulse (30 ms), waiting time (3 s), and N_2_ purge (15 s). N_2_ served as both the carrier gas and purge gas at a flow rate of 30 sccm. The precursors used were purchased from J&K Scientific Ltd., China. The thicknesses of ZnO nanomembranes in the composites were tuned by changing the number of ALD cycles: 20, 100, and 300 cycles (shorted as GAZ20, GAZ100 and GAZ300). Then, the samples were annealed in tube furnace at 700 °C for 120 min in N_2_ atmosphere. For comparison, pure GA was also annealed in tube furnace at 700 °C for 120 min in N_2_ atmosphere.

### Microstructural Characterizations

The morphologies and microstructures of the GAZ composites were examined using scanning electron microscopy (SEM, Zeiss Sigma) and transmission electron microscope (TEM, Nova NanoSem 450). The X-ray diffractometer (XRD) patterns were recorded using a Bruker D8A Advance XRD with Cu Kα radiation (*λ* = 1.5405 Å). The composition of GAZ composites was tested by energy dispersive spectroscopy (EDS) attached to SEM.

### Electrochemical Measurements

The electrochemical tests were accessed on a CR2016 coin cell with lithium metal acting as both the counter and reference electrode. The working electrode was composed of 80 wt.% active material (i.e., GAZ composites), 10 wt.% conductive additive agent (Super P), and 10 wt.% binder (polyvinylidene difluoride in *N*-methyl-2-pyrrolidone (NMP)). The electrolyte used was a solution of 1 M LiPF_6_ dissolved in ethylene carbonate/diethyl carbonate (EC/DEC, 1:1 *v*/*v*). The cells were assembled in an argon-filled glove box (H_2_O, O_2_ < 1 ppm). Galvanostatic measurements were performed on a battery testing system (LAND CT2001A) in the voltage range of 0.01–3 V. The current rates used were based on the total mass of the electrode. Cyclic voltammetry (CV) tests were also carried out at a scan rate of 0.1 mV s^−1^ from 0.001 to 3 V using a Zennium/IM6 electrochemical workstation.

## Results and Discussion

**Fig. 1:**
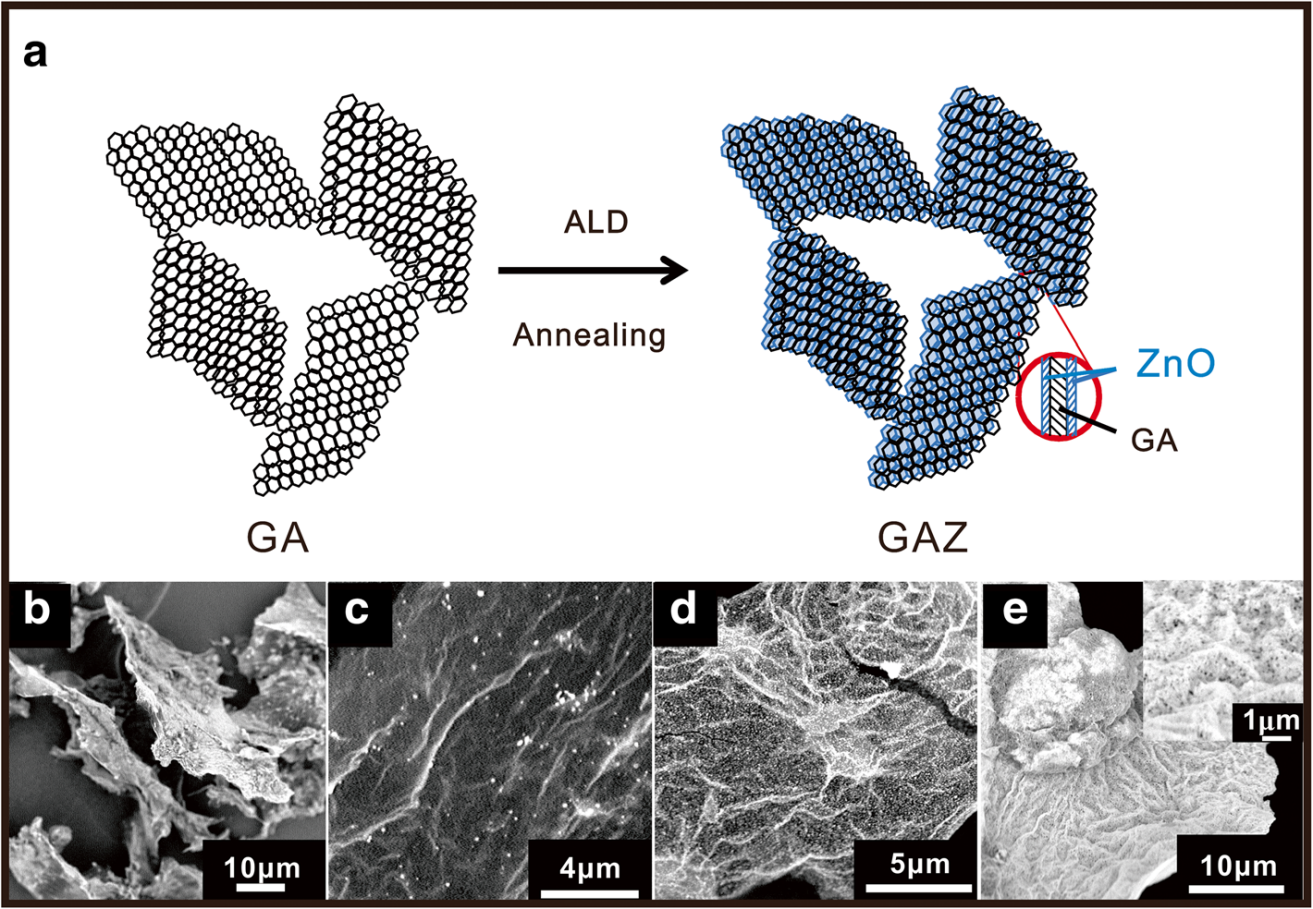
**a** Fabrication schematic of GAZ composites. SEM images of **b** GA, **c** GAZ20, **d** GAZ100, and **e** GAZ300. The inset in **e** is enlarged SEM image of GAZ300

The fabrication schematic of GAZ composites is depicted in Fig. 1a. GA was synthesized by a template-free, freeze-drying strategy. Then, ALD was utilized to decorate GA surface with ZnO nanomembranes. The morphology and microstructure of GA and GAZ were demonstrated by means of SEM. Figure 1b clearly shows that GA was made up of graphene nanosheets. Figure 1c–e displays the microstructural similarities and differences in GAZ composites with the increasing number of ALD cycles. One can see that ZnO nanomembranes are well deposited on the GA surfaces, yet the surface coverages are quite different. The graphene layers in GAZ20 are not completely coated by ZnO nanomembranes (Fig. 1c). The ZnO was distributed as dots/islands on GA surface due to the lack of reactive sites or functional groups on GA surface [25]. When the number of ALD cycles is increased to 100, the surface of GA is entirely decorated with ZnO nanomembrane consisting of small nanoparticles, as shown in Fig. 1d. Figure 1e and the corresponding enlarged image in the inset demonstrates that a thick and dense ZnO nanomembrane was formed with more ALD cylces. SEM images in Fig. 1 demonstrate that the ZnO coverage on GA surface increases correspondingly with the increasing ALD cycles.

**Fig. 2:**
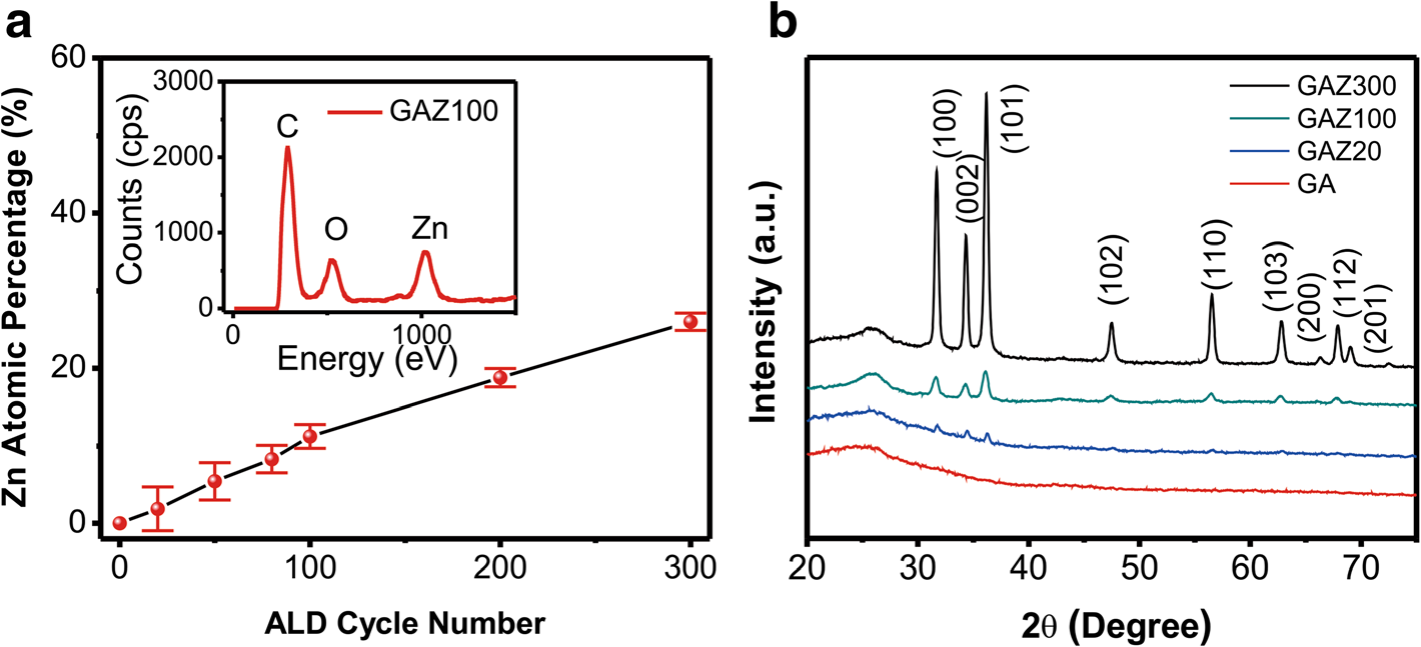
**a** Atomic percentage of Zn atoms in GAZ composite. The inset is the EDS result of GAZ100. **b** XRD patterns of GA and GAZ composites with different ALD cycles

EDS analyses were used to determine the chemical compositions of GAZ composites. As shown in the inset of Fig. 2a, the existence and atom percentages of O and Zn indicate that ZnO nanomembranes were successfully decorated on the GA surface which is consistent with the SEM images. The atomic percentage of Zn in GAZ as s function of ALD cycles is illustrated in Fig. 2a, and an obvious increase of Zn concentration is observed, which indicates the composition of the composites can be easily tuned by changing ALD cycles. To investigate the crystal structure of these composites, the composites were characterized by XRD and the results are shown in Fig. 2b. For GAZ300 and GAZ100, the characteristic diffraction peaks of ZnO (100), (002), (101), (102), (110), (103), (112), and (201) are clearly exhibited in XRD patterns (PDF#36–1451) [21], suggesting that ZnO nanomembranes coated on GA surfaces can maintain the hexagonal wurtzite structure. However, very weak diffraction peaks can be distinguished in GAZ20 because the content of ZnO is too low. With the increasing number of ALD cycles, the characteristic peak of ZnO is more obvious due to higher ZnO concentration. The experimental results in Fig. 2 further prove that the composition of the composite is successfully tuned by changing the ALD cycles; thus, the influence of the composition on device performance can be easily probed.

**Fig. 3:**
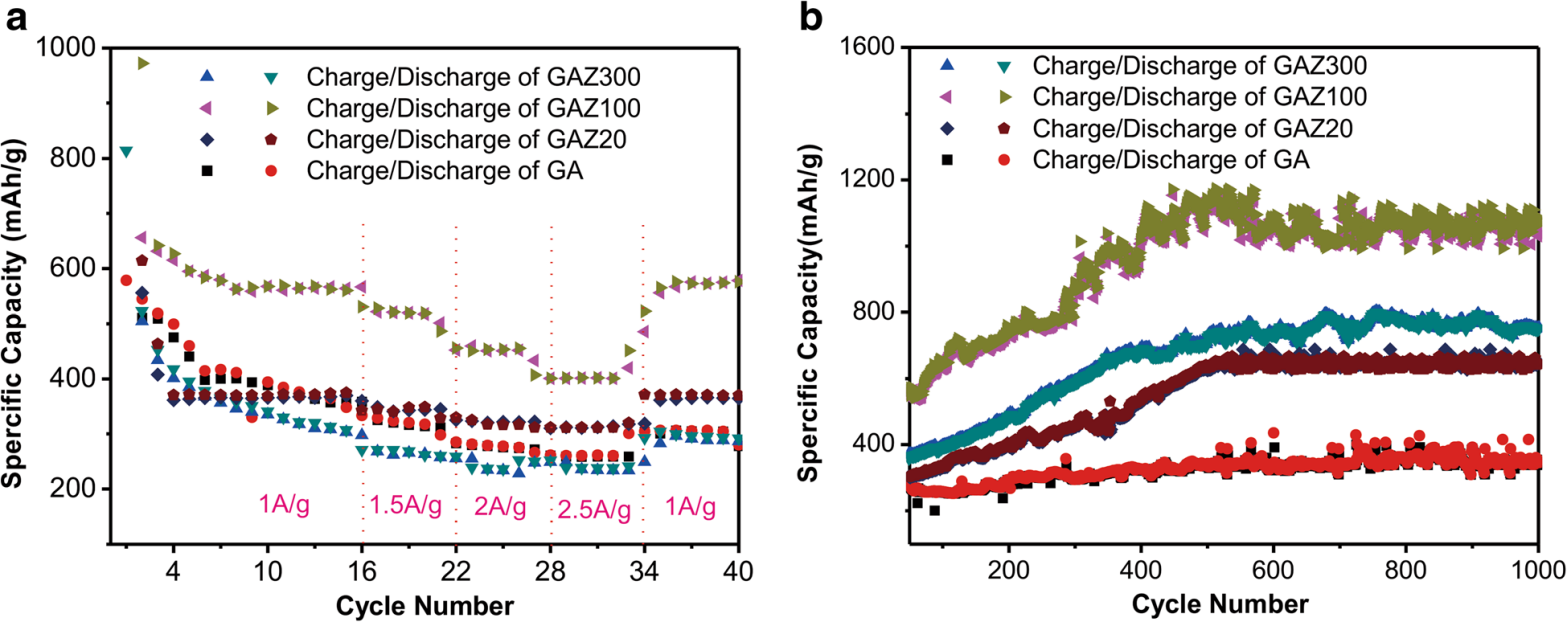
**a** Rate performance of pure GA and GAZ composite with different ALD cycles. **b** Cycle performance of pure GA and GAZ composite with different ALD cycles. A high current density of 1000 mA g^−1^ was used in the experiment

The rate performance of pure GA and GAZ composites with different ALD cycles was evaluated at various current densities (1000–2500 mA g^−1^ as depicted in Fig. 3a). Both current density and capacity were calculated based on total mass of the electrode. GA20 shows stable capacity at even high current density (2.5 A g^−1^). As the number of ALD cycles increases to 100, the GAZ100 electrode shows better rate performance. As the current density increases to 1500, 2000, and 2500 mA g^−1^, the GAZ100 electrode exhibits the capacity of 520, 450, and 400 mAh g^−1^, respectively. When the current density returns back to 1000 mAh g^−1^, the GAZ100 electrode recovers the initial reversible capacity of 600 mAh g^−1^. The excellent rate performance is attributed to the good conductivity, porous structure, and mechanical flexibility of GA, which facilitate the fast *e*^−^/Li^+^ transport in the composite electrode and alleviate ZnO pulverization. One may note that the initial discharge capacity of pure GA is higher than its theoretical capacity. The extra capacity was attributed to the decomposition of electrolyte to form the solid electrolyte interphase (SEI) layer [31]. When the number of ALD cycles increases to 300, the GAZ300 delivers lower capacity and shows worse rate performance than GAZ100. Therefore, rate performance is not positively correlated with the number of ALD cycles. We infer that the low content of ZnO in GAZ20 leads to the lower charge-discharge capacity. As ALD cycles increases to 300, the resistance of the composite increases correspondingly, and the thicker ZnO nanomembranes entirely covered the GA surface, which is not beneficial for electrolyte penetration and lithium-ion transmission. In addition, the volume change of thicker ZnO cannot be well relaxed in GAZ300. As a result, the rate performance of GAZ300 deteriorates although it possesses a higher ZnO content.

To investigate the specific capacity in more detail, we took a long-cycle test of pure GA and GAZ composites at current rate of 1000 mA g^−1^ for 1000 cycles after the rate performance test, and the results are illustrated in Fig. 3b. The specific capacity of GAZ composites obviously increased from 50th to 500th cycles. It is noted that capacity increases from 580 mAh g^−1^ to 1200 mAh g^−1^ for GAZ100, from 450 to 700 mAh g^−1^ for GAZ300, from 300 to 600 mAh g^−1^ for GAZ20. Correspondingly, the highest areal capacity of GAZ100 is 0.61 mA/cm^2^, which is higher than those of GAZ20 (0.31 mAh/cm^2^) and GAZ300 (0.35 mAh/cm^2^). However, the capacity of pure GA in the long cycle only shows a small capacity increase, and ZnO also did not show obvious capacity increase in previous research [7, 23, 32]. This indicates that the capacity increase in GAZ composites should result from the co-effect of ZnO and GA components. Such a capacity increase phenomenon in cycling process has been observed in anodes made from many metal oxides [9, 33–37] and was ascribed to the formation of reversible polymer layer due to activated electrolyte degradation [9]. Previous literatures [16, 38, 39] have proved that the layer can effectively store the Li-ions and the capacity is therefore enhanced.

**Fig. 4:**
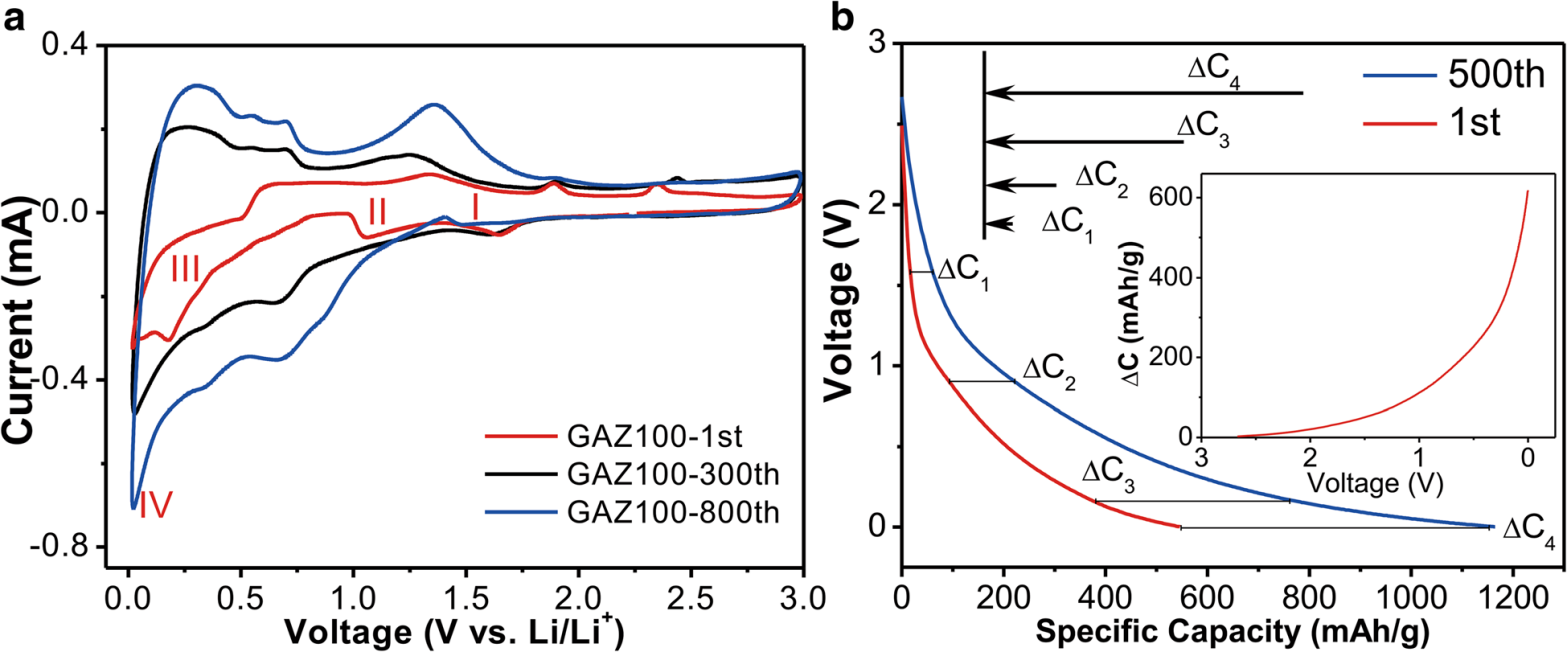
**a** CV for GAZ100 after different charge/discharge cycles. **b** Selected discharge voltage profiles. Red and blue lines illustrate the discharge profiles of the 1st cycle and 500th cycle, respectively. Inset illustrates the difference of capacities between the 1st and the 500th cycles, as the function of discharge voltage

To further investigate the capacity increase phenomenon, we carried out CV test of GAZ100 electrode. Figure 4a illustrates the CV profiles of the GAZ100 electrode of the 1st, 300th, and 800th cycles, which were recorded with the potential window of 0.01–3.0 V at the scanning rate 0.1 mV s^−1^. In the first cycle, four cathodic peaks located at 1.6 V (I), 0.9 V (II), 0.2 V (III), and 0.06 V (IV) were observed. The peak positioned at 1.6 V (I) could be associated with the formation of the SEI layer [19, 40]. The peaks observed at 0.9 (II) and 0.2 V (III) correspond to the reduction of ZnO to Zn (ZnO + Li^+^ + 6e^−^ → Zn + Li_2_O) and the alloying process (xLi + Zn → Li_x_Zn), respectively [19, 32, 41–43]. In addition, the strong reduction peak closed to 0.06 V (IV) is related to the lithiation process of GA [15, 44]. Compared with the first cycle, the cathodic peaks at 1.6 V (I) after 300 cycles still exists indicating that the formation of SEI layer still occurred in subsequent long cycles. However, the peak at 1.6 V (I) disappears after 800 cycles, indicating the stable formation of SEI layers. The reduction peaks at 0.9 (II) and 0.2 V (III) shift to 0.62 and 0.3 V, respectively, after 300 and 800 charge/discharge cycles. On the basis of the aforementioned discussion, we attributed this shift to the reduction reactions of ZnO to Zn accompanied by the formation of the polymer layer [9, 45, 46], as will be discussed later. As for the anodic curve, five peaks at 0.2, 0.5, 1.3, 1.7, and 2.3 V are observed. The oxidation peaks at 0.2, 0.5, and 1.3 V correspond to the multi-step dealloying process of the Li_x_Zn alloy to form Zn, and the peaks at 1.7 and 2.3 V correspond to the oxidation of Zn to generate ZnO [7, 47]. In subsequent cycles, it can be clearly seen that all these anodic peaks shift to higher voltages. It indicates the faster electron transport or slower deintercalation of lithium ion in GAZ100 anode in subsequent cycles. However, the expansion/contraction of ZnO in charge/discharge cycles should cause relatively worse contact with GA, resulting in slower electron transport. Thus, the observed peak shift to higher voltage should be mainly ascribed to the slower deintercalation of lithium ion. Previous literature has demonstrated that the formation of the polymer layer would increase the interfacial resistance and the deintercalation of lithium ion would be hindered [48]. In addition, it is worth noting that the integrated area of anodic and cathodic peaks increases with the cycles (Fig. 4a), which is consistent with the increased capacity shown in Fig. 3b.

Figure 4b demonstrates the selected discharge voltage profiles of the 1st and the 500th cycles of GAZ100. The corresponding capacity increment is shown in the inset of Fig. 4b. It is demonstrated that most capacity increment was gained at 0.02–0.9 V. According to the CV illustrated in Fig. 4a, the discharge process can be divided into four stages based on the four voltage ranges of 3.0–1.6, 1.6–0.9, 0.9–0.2, and 0.2–0.06 V, corresponding to the formation of SEI layer, reduction of ZnO to Zn, alloying process accompanied by the formation of the polymer layer, and lithiation process of GA, respectively. As described in Fig. 4b, ∆C_1_, ∆C_2_, ∆C_3_, and ∆C_4_ are the capacity increments of the respective voltage ranges from the 1st to 500th cycle. The total capacity increase (from the 1st to the 500th cycles, 589.1 mAh g^−1^, ∆C_4_) consists of the growing capacity from the SEI layer formation (44.4 mAh g^−1^, ∆C_1_), ZnO reduction to Zn (80.4 mAh g^−1^, ∆C_2_ − ∆C_1_), the alloying process of Zn and Li (258 mAh g^−1^, ∆C_3_ − ∆C_2_), and the GA lithiation process (206.3 mAh g^−1^, ∆C_4_ − ∆C_3_). Obviously, the major capacity increase (∆C_3_ − ∆C_2_) mainly occurred in the low potential ranges, where the polymer layer may form, as described in previous literatures [49, 50]. In addition, we consider that the gradual exposure of active material (i.e., GAZ composites) to electrolyte after charge/discharge cycles may also partially contribute to the capacity increase (∆C_4_ − ∆C_3_).

The morphology of GAZ100 electrode after 500 cycles was investigated in detail to prove the stability of the electrodes. Typical TEM image of GAZ100 electrode after 500 charge/discharge cycles is shown in Additional file 1: Figure S1, and the crystal lattice of ZnO can be clearly observed. The TEM results shown in Additional file 1: Figure S1 indicate that the ZnO nanocrystals did not crack after 500 cycles, suggesting a stable performance of current composite [23].

## Conclusion

In summary, GAZ composites were easily synthesized via ALD. The composition of GAZ could be finely tuned by changing the number of ALD cycles. Characterization demonstrates that the electrodes made from composites exhibit better rate performance and higher capacity because the composite combines the excellent conductivity and flexibility of GA with high specific capacity of ZnO nanomembranes. A remarkable capacity increase with cycling (from 580 mAh/g to 1200 mAh/g for GAZ100 electrode) was observed in GAZ composites. Detailed electrochemical analyses suggest that the phenomenon is caused by the formation of polymer layer at low voltage region, which can storage more lithium so that the reversible capacity was higher. The convenient fabrication process and high reversible capacity of the GAZ composites make them promising anode materials for future LIBs.

## Additional file

Additional file 1: **Figure S1.**. (a) HRTEM image of GAZ100 after 500 discharge/charge cycles. (b) SAED pattern of GAZ100 after 500 discharge/charge cycles. (DOCX 408 kb)

## Abbreviations

ALD: Atomic layer deposition
CV: Cyclic voltammetry
DEC: Diethyl carbonate
EC: Ethylene carbonate
EDS: Energy dispersive spectroscopy
GA: Graphene aerogel
GAZ: Zinc oxide/graphene aerogel
GO: Graphene oxide
LIBs: Lithium-ion batteries
NMP: *N*-Methyl-2-pyrrolidone
PFOES: Perfluorooctyltriethoxysilane
SEI: Solid electrolyte interphase
SEM: Scanning electron microscopy
TEM: Transmission electron microscope
XRD: X-ray diffractometer
ZnO: Zinc oxide
