# Supplementary material for: Cycling-Induced Capacity Increase of Graphene Aerogel/ZnO Nanomembrane Composite Anode Fabricated by Atomic Layer Deposition
Source: Nanoscale Res Lett. 2019 Feb 28;14:69. doi: 10.1186/s11671-019-2900-7 (PMC6395466; doi:10.1186/s11671-019-2900-7)
Supplement: Supplementary file 1 — Figure S1.. (a) HRTEM image of GAZ100 after 500 discharge/charge cycles. (b) SAED pattern of GAZ100 after 500 discharge/charge cycles. (DOCX 408 kb) [file 11671_2019_2900_MOESM1_ESM.docx]

Supplementary Material for

# Cycling induced capacity increase of graphene aerogel/ZnO nanomembranes composite anode fabricated by atomic layer deposition

*Dingrun Wang^1,§^, Yalan Li^1,§^, Yuting Zhao^1^, Qinglei Guo,^1^ Siwei Yang^2^, Guqiao Ding^2^, YongFeng Mei^1^*, *Gaoshan Huang^1^^[[1]](#footnote-1)^*,*

*1 Department of Materials Science, Fudan University, Shanghai 200433, P. R. China*

*2 State Key Laboratory of Functional Materials for Informatics, Shanghai Institute of Microsystem and Information Technology, Chinese Academy of Science, Shanghai 20050, P. R. China*


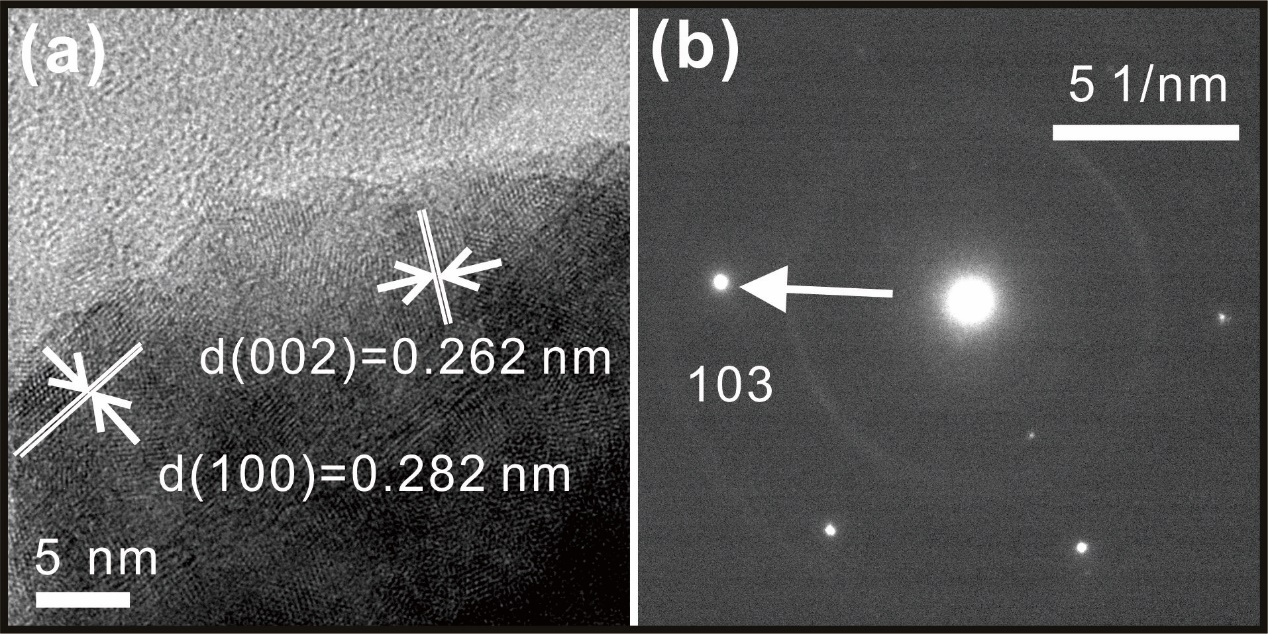


Fig. S1 (a) HRTEM image of GAZ100 after 500 discharge/charge cycles. (b) SAED pattern of GAZ100 after 500 discharge/charge cycles.

1. * Corresponding author: [gshuang@fudan.edu.cn](mailto:gshuang@fudan.edu.cn)

   ^§^ These two authors contributed equally [↑](#footnote-ref-1)
